# Large venous-arterial PCO_2 _is associated with poor outcomes in surgical patients

**DOI:** 10.1186/cc10210

**Published:** 2011-06-22

**Authors:** JM Silva, AMRR Oliveira, VPl Maia, AMP Ferreira, DO Toledo, E Rezende, LMS Malbouisson

**Affiliations:** 1 HSPE, São Paulo - SP, Brazil

## Introduction

This study evaluated whether a large venous-arterial CO_2 _gap (PCO_2 _gap) during the preoperative period is associated with poor surgical outcome.

## Methods

A prospective observational study that included high-risk surgical patients who were 18 years of age or older. Palliative surgery, Child B and Child C cirrhosis, and class IV heart condition patients or ejection fraction <30% were excluded. The patients were divided into two groups: wide [P(v-a)CO_2_] versus narrow [P(v-a)CO_2_]. In order to determine the best value to discriminate hospital mortality, the receiver operating characteristic curve was used for the [P(v-a)CO_2_] values collected during the preoperative period, and the most accurate value was chosen as a cut-off to define the groups.

## Results

The study included 66 patients. The preoperative [P(v-a)CO_2_] value that best discriminated hospital mortality was 5.0 mmHg, area = 0.73. Preoperative patients with [P(v-a)CO_2_] of more than 5.0 mmHg presented a higher hospital mortality (36.4% vs. 4.5%, *P *= 0.004), higher prevalence of circulatory shock (56.8% vs. 22.7%, *P *= 0.01) and acute renal failure in the postoperative period (27.3% vs. 4.5%, *P *= 0.02), and longer length of hospital stays (20.0 (14.0 to 30.0) vs. 13.5 (9.0 to 25.0) days, *P *= 0.01). The groups did not present any differences regarding demographic and physiological data.

## Conclusion

The PCO_2 _gap values of more than 5.0 mmHg in the preoperative period were associated with worse postoperative outcome.

